# Benefit of Treatment Individualization in Patients with Chronic Hepatitis C Receiving Peginterferon Alfa-2a and Ribavirin in a Large Noninterventional Cohort Study

**DOI:** 10.1371/journal.pone.0134839

**Published:** 2015-07-31

**Authors:** Wolf Peter Hofmann, Stefan Mauss, Thomas Lutz, Andreas Schober, Klaus Böker, Gero Moog, Axel Baumgarten, Heike Pfeiffer-Vornkahl, Ulrich Alshuth, Dietrich Hüppe, Heiner Wedemeyer, Michael P. Manns, Eckart Schott

**Affiliations:** 1 Practice of Gastroenterology, Berlin, Germany; 2 Center for HIV and Hepatogastroenterology, Düsseldorf, Germany; 3 Infektiologikum, Frankfurt, Germany; 4 Practice of Hepatology, Göttingen, Germany; 5 Practice of Hepatology, Hannover, Germany; 6 Practice of Gastroenterology, Kassel, Germany; 7 Center of Infectious Diseases, Berlin, Germany; 8 Institute for Clinical Research and Statistics, Offenbach, Germany; 9 Virology, Roche Pharma AG, Grenzach-Wyhlen, Germany; 10 Center of Gastroenterology, Herne, Germany; 11 Department of Gastroenterology, Hepatology, and Endocrinology, Medizinische Hochschule Hannover, Hannover, Germany; 12 Dept. of Hepatology and Gastroenterology, Charité Universitätsmedizin Berlin, Campus Virchow Klinikum, Berlin, Germany; National Institutes of Health, UNITED STATES

## Abstract

**Background and Aims:**

Individualization of treatment with peginterferon alfa and ribavirin in patients with chronic hepatitis C showed benefit in controlled trials and was implemented in treatment guidelines to increase response rates and to reduce side effects and costs. However, it is unknown whether individualization was adopted in routine daily practice and whether it translated into improved outcomes.

**Methods:**

From a large noninterventional cohort study, clinical and virologic response data of 10,262 HCV patients who received peginterferon alfa-2a and ribavirin between 2003-2007 and 2008-2011 were analyzed. To account for treatment individualization, a matched-pair analysis (2,997 matched pairs) was performed. Variation in treatment duration and dosing of ribavirin were analyzed as indicators for individualization.

**Results:**

Sustained virological response (SVR) rates were similar between 2003-2007 and 2008-2011 (62.0% vs. 63.7%). Patients with comorbidities were more abundant in the later period, (44.3% vs. 57.1%). The subsequent matched-pair analysis demonstrated higher SVR rates in the 2008-2011 period (64.3%) than in the 2003-2007 period (61.2%, p=0.008). More patients received abbreviated or extended treatment regimens in the later than the earlier period as an indicator of treatment individualization. To the same end, ribavirin doses were higher in the later period (12.6 versus 11.6 mg/kg/day). Factors independently associated with SVR included HCV genotype, low baseline viral load, younger age, route of infection, absence of concomitant diseases, lower APRI score, normal gamma-GT, higher ribavirin doses, no substitution for drug abuse, treatment duration, and treatment in the 2008-2011 period.

**Conclusions:**

Treatment individualization with peginterferon alfa and ribavirin was implemented in daily routine between 2003-2007 and 2008-2011, SVR rates improved in the same period. These findings may be most relevant in resource-limited settings.

## Introduction

Chronic hepatitis C virus infection (CHC) causes progressive liver disease. The risk of developing liver cirrhosis is estimated at 5–30% after 20–30 years of infection [1,2]. A sustained virologic response (SVR) after a course of antiviral therapy is generally accepted as a surrogate marker for cure. SVR prevents the progression of liver disease, reduces the risk of HCC, improves liver-related and overall mortality, and eliminates the risk to spread HCV infection [3,4].

Prior to licensing of the first direct-acting antiviral agents (DAA) in 2011, treatment with pegylated interferon (pegIFN) alfa-2a or alfa-2b and ribavirin was the standard-of-care for patients with CHC for nearly one decade [5]. According to initial treatment algorithms, patients infected with HCV genotype 1 or 4 were treated for 48 weeks and those infected with HCV genotype 2 or 3 were treated for 24 weeks, resulting in SVR rates ranging from 42% to 52% and 76% to 82%, respectively [6–9]. More recently, host and viral factors associated with treatment response were identified and were used to tailor treatment according to a patient’s individual profile. Host factors include age, gender, ethnicity, weight, associated liver disease, insulin resistance, adherence to therapy, and genetic polymorphisms such as the *IL28B* genotype [10,11]. Viral factors include the HCV genotype, the pretreatment HCV RNA level and the on-treatment virologic response at weeks 4 (RVR, rapid virological response) and 12 (EVR, early virological response) [12].

Several randomized controlled trials were conducted in the past decade to individualize treatment algorithms for chronic hepatitis C in particular with regard to treatment duration [12]. Abbreviated treatment regimens should ideally reduce the rate of adverse side effects and costs without compromising antiviral efficacy. Extended treatment durations aim at increasing SVR rates in selected patients who are slow responders to treatment [13–15]. Indeed, patients with genotype 1 and low baseline viral load who achieve RVR can shorten treatment to 24 weeks without reducing efficacy [16–18]. Treatment extension to 72 weeks in genotype 1 patients is considered in subgroups of patients without complete EVR [19–21]. Selected genotype 2 or 3 patients who achieve RVR and who exhibit additional favorable prognostic host factors may likewise be treated for shorter durations (12–16 weeks) [22–25]. Genotype 2 or 3 patients who fail to eliminate HCV RNA by week 4 may instead benefit from extending treatment duration to 48 weeks [26].

Dosing of ribavirin is another important factor for individualization as higher doses improve SVR rates and reduce virologic relapse rates (27, 28). Administration of weight-based ribavirin (ranging from 800 mg to 1400 mg per day in genotype 1 patients) is superior to fixed dosed ribavirin (800 mg). Higher doses of up to 15 mg per kg are more effective but are also associated with higher rates of adverse effects (29, 30).

Noninterventional cohort studies fill the gap between highly controlled randomized trials and the reality of everyday practice. They usually enroll a broader population of patients than randomized controlled trials, including those who are older and have comorbidities. Furthermore, they provide information about adherence of physicians to practice guidelines.

Results of several noninterventional cohort studies demonstrated that treatment efficacy in everyday practice is generally comparable to clinical trials of HCV treatment [27, 28]. However, it is unknown whether individualization of treatment regimens has been adopted by physicians and has led to improved SVR rates in daily practice [29].

## Patients and Methods

This analysis derives from three consecutive German multicenter noninterventional studies in patients with CHC receiving pegIFN alfa-2a and ribavirin, involving 432 physicians/institutions throughout Germany (369 in office based practice and 63 in hospital settings).

Patients were included in the first study from March 2003 to February 2006. The second study recruited patients from March 2006 to December 2008 and the third study started in January 2009. The last patient entered this study in October 2011, and the last data entry was in December 2013. A total of 16,359 patients were included in these noninterventional studies. The present analysis was conducted in January 2013 with 12,081 previously untreated patients without HIV or HBV coinfection and without previous solid organ transplantation of whom documentation was complete.

The recommended treatment duration for patients with genotypes 2 and 3 infection was 24 weeks until 2010, when German guidelines [30] were revised and recommended 16 weeks of treatment in patients who achieved RVR and 48 weeks of treatment in patients who did not achieve RVR. For genotype 1, 4, 5, and 6 infections the recommended treatment duration was 48 weeks initially. In 2007 abbreviation of the treatment duration to 24 weeks was approved for pegIFN alfa-2a for patients with genotype 1 infection and low baseline viremia (≤ 800.000 IE/ml) or with genotype 4 who achieved RVR. The treatment period was followed by an observational period of 24 weeks. The recommended dosage of pegIFN alfa-2a was 180 μg once weekly in combination with ribavirin according to the SPCs of manufacturers.

Inclusion criteria in the present cohort were age of at least 18 years, quantifiable HCV RNA (baseline HCV-RNA was not available in 439 out of 16,359 patients), compensated liver disease, and written informed consent. RVR was defined as HCV RNA negativity at week 4 with a window from day 23–33 of treatment. SVR was defined as non-detectable HCV RNA 24 weeks after completion of the treatment period. Virological failure was defined as <2 log decline of HCV RNA from baseline at week 12.

Data were obtained on structured questionnaires. Screening data included demographic data, history of HCV infection, and concomitant diseases. On-treatment information about virological response and drug safety was collected using online data entry. The studies represent an unselected cohort in a real-life setting including a significant fraction of all patients treated for chronic hepatitis C monoinfection in Germany.

Because of limited numbers of liver biopsies, the degree of liver fibrosis was estimated by APRI (aspartate aminotransferase—platelet ratio index) using pre-treatment laboratory data [31].

For comparison of the evolvement of treatment outcomes two periods were compared: an earlier period before the recommendation of treatment individualization (2003–2007) and a later period after recommendation of treatment individualization (2008–2011).

The weight-based ribavirin dose was calculated as follows: cumulative ribavirin dose divided by the number of days of treatment and the body weight of the patient per kilogram.

### Ethics statement

Studies were announced to health authorities and ethical committees according to German drug law. The study was approved by health authorities and the ethical committee Ethik-Kommission der Ärztekammer Westfalen-Lippe, Münster, Germany. This study and all related studies for this drug are registered in the national register for non-interventional studies at vfa (Verband Forschender Arzneimittelhersteller e.V., Berlin, Germany). Written informed consent was obtained from all patients.

### Study cohort

The entire cohort consists of 16,359 HCV patients who received pegIFN alfa-2a and ribavirin between 2003–2007 (n = 8,563) and 2008–2011 (n = 7,796).

#### Intention-to-treat cohort (ITT)

2,058 patients were excluded because they were not treatment naïve, 803 were excluded due to HIV co-infection, 748 were excluded due to HBV co-infection, and 16 patients were excluded because they had previously received an organ transplant. The entire naïve ITT cohort therefore consisted of 12.801 treatment-naïve patients infected with HCV, 6,643 of whom were treated in the 2003–2007 period while 6,158 patients were treated in the 2008–2011 period.

#### Per protocol cohort (PP)

The per protocol cohort consisted of 10,262 patients, excluding patients whose treatment was discontinued for other reasons than poor tolerability, concomitant diseases, or virological non-response, i.e. mainly due to non-compliance or patients’ request as well as loss to follow-up. 5,386 patients from the PP cohort were treated in the 2003–2007 period, and 4,876 patients were treated in the 2008–2011 period.

#### Matched-pairs analysis from the PP cohort (MP-PP)

To account for differences regarding patient characteristics and important predictive baseline factors in the earlier and later treatment period, a matched-pairs analysis was performed for the PP cohort. Matched pairs were formed by matching age within 5 years, the HCV genotype, baseline viral load (low or high viral load with best cut-off of ≤ 470.000 IE/ml, see below), APRI-Score (< 0.5, 0.5–< = 1.5, 1.5–< = 2, > 2), possible route of infection (intravenous drug abuse, blood or blood products, other/unknown), presence of concomitant diseases, baseline gamma-GT (within normal range (male < 66 U/l; female: < 39 U/l) vs. elevated), and substitution for intravenous drug abuse. A total of 2,997 matched pairs (PP MP) were created with the above mentioned matching criteria.

#### Matched-pairs analysis from PP completer cohort (MP-PP completer)

In order to determine whether treatment individualization was implemented in the later period, patients who discontinued treatment before the planned treatment termination were excluded from the PP matched-pairs completer cohort. This data set should be better suited to analyze whether treatment individualization did indeed take place in the later period as factors confounding the treatment duration were eliminated. A total of 2,439 of the patients included in this cohort were treated in the 2003–2007 period, and 2,475 patients were treated in the 2008–2011 period.

#### Statistical Analysis

The statistical analysis was descriptive to reflect the clinical routine as intended by the clinicians. Summary statistics (mean, median, standard deviation, 25^th^ percentile, 75^th^ percentile, minimum, maximum, number of values) or frequencies and proportions were assessed depending on the scale level of the data.

For the continuous predictive factors age and baseline HCV RNA receiver operating characteristic (ROC) analyses estimated the best cut-off point for SVR. The cut-off points found for the continuous variables were used to generate the corresponding categorial variables. The cut-off point for age was determined as < 45 / ≥ 45 years. The cut-off for baseline viral load was determined as < 470.000 IU/ml versus / ≥ 470.000 IU/ml. Route of infection was categorized as transfusion/blood products, illicit drugs, or other/unkown. Further categorical variables were gender, concomitant disease (yes/no), substitution for opioid abuse (yes/no), elevated gamma-GT (yes/no), and APRI score (< 0.5; 0.5–≤ 1.5; 1.5–≤2; > 2). An univariate analysis of these factors was performed initially followed by multivariate analysis of all factors showing significant associations in the univariate analysis (p≤0.05).

Differences in baseline clinical characteristics and safety and efficacy data were compared using univariate and multiple logistic regression analyses including odds ratio and 95% confidence interval.

All statistical analyses were based on 2-sided hypothesis tests. Analyses were carried out using SPSS 12.0.2 (SPSS Inc., Chicago, Illinois, USA), Testimate, version 6.4.27 (Institute for Data Analysis and Study Planning, Gauting/Munich, Germany) and Matched Version 1.1. (Institut für Medizinische Statistik und Dokumentation, Erlangen).

## Results

### Characterization of the study cohort

**Fig 1:**
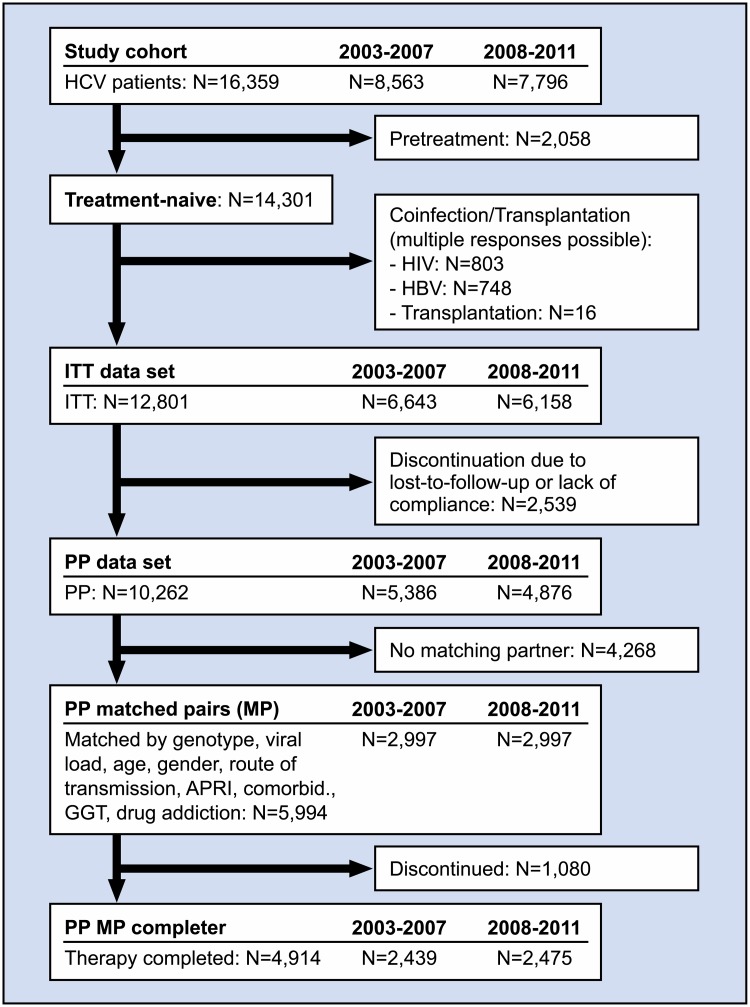
Patient disposition. APRI, aspartate aminotransferase (AST) platelet ratio index; GGT, gamma-glutamyl transferase; HBV, hepatitis B virus; HIV, human immunodeficiency virus; ITT, intent to treat; MP, matched pairs; PP, per protocol

Between 2003 and 2011 a total of 16,359 patients were included into the study, 8,563 patients were treated in the 2003–2007 period, and 7,796 patients were treated in the 2008 to 2011 period (Fig 1). 9,609 patients were infected with HCV genotype 1, 1,116 and 4,964 patients were infected with HCV genotypes 2 and 3, respectively, and 605 patients were infected with HCV genotype 4. Few patients were infected with HCV genotypes 5 (n = 30) or 6 (n = 35). 63.5% of patients were male, and the median age was 42 years. 84.1% of patients were of Caucasian ethnicity. The possible route of infection reported most frequently by the physician was intravenous drug abuse in 42.3% of patients, followed by treatment with blood or blood products in 13.7% of patients. The possible route of infection was unknown/other in 44.0% of patients. 56.7% of patients had concomitant diseases. 12.8% suffered from psychiatric disease, 8.4% suffered from cardiovascular disease, and 4.0% and 3.0% suffered from diabetes and pulmonary disease, respectively. 22.8% of patients self-reported former or current intravenous drug abuse and 12.1% of patients were on opioid substitution treatment.

In the ITT cohort SVR rates were 50.3% in the 2003–2007 period and 50.5% in the 2008–2011 period.

Further analyses were performed in the PP cohort. SVR rates were also not statistically different in the PP cohort between the early and late treatment period (62.0% versus 63.7%). Likewise SVR rates did not differ for patients infected with HCV genotype 1 (52.0% vs. 55.6%), genotype 2 (73.9% vs. 76.2%), genotype 3 (79.3% vs. 77.6%), or genotype 4 (56.9% vs. 54.4%). The percentage of relapsers (13.6% vs. 12.9%) and virologic non-responders (24.4% vs. 23.3%) also did not differ.

Treatment was terminated due to virologic non-response in 11.2% of patients in the earlier treatment period and in 13.1% of patients in the later treatment period and due to poor tolerability in 6.3% and 4.0%, respectively. Patients terminating the treatment for other reasons (incompliance, poor tolerability, non-compliance, patients’ request, or loss to follow-up) were excluded form the PP cohort but are shown for the ITT cohort (Table 1).

**Table 1:** Baseline demographics, virological responses, treatment discontinuations as well as treatment duration and mean ribavirin dose in the two treatment periods 2003–2007 and 2008–2011 in the ITT and PP cohorts.

	ITT				PP			
	2003–2007	2008–2011	p-value	total	2003–2007	2008–2011	p-value	total
**Number of patients (n)**	6643	6158		12801	5386	4876		10262
**Age (Yrs, mean)**	41.6	41.8	n.s.	41.7	42.5	42.7	n.s.	42.6
**Gender (male, %)**	60.4	63.3	**	61.8	58.5	60.8	**	59.6
**BMI (kg/m², mean)**	25.1	25.5	***	25.3	25.2	25.6	***	25.4
**Ethnicity, Caucasian (%; adjusted by unknown)** ^†^	74.6	96.2	*	85.0	73.6	96.0	n.s.	84.3
**APRI Score (%)**			n.s.				n.s.	
< 0.5	44.5	44.5		44.5	44.3	44.0		44.2
0.5-< = 1.5	41.7	42.0		41.8	41.8	42.2		42.0
1.5-< = 2	4.8	4.7		4.7	4.8	4.8		4.8
>2	8.9	8.9		8.9	9.0	9.0		9.0
**Baseline GGT (IU/ml, mean)**	80	93	**	86	81	90	*	85
**Route of infection (%):**			***				***	
Transfusion/blood products	16.8	10.5		13.8	18.5	11.8		15.3
Intravenous drug abuse	40.7	45.9		43.2	36.7	42.0		39.2
Other / unknown	42.5	43.7		43.1	44.8	46.2		45.4
**Concomitant diseases (%):**	44.3	58.0	***	50.9	44.3	57.1	***	50.4
Psychiatric	8.9	14.6	***	11.6	8.5	14.4	***	11.3
Cardiovascular	7.6	8.5	n.s.	8.0	8.5	9.4	n.s.	8.9
Diabetes	3.5	3.8	n.s.	3.6	3.8	4.1	n.s.	3.9
Pulmonary	2.6	3.2	n.s.	2.9	2.7	3.5	*	3.1
Former iv drug abuse	15.1	30.4	***	22.5	13.5	27.1	***	20.0
Opioid substitution treatment	7.4	17.4	***	12.2	6.4	16.1	***	11.0
**Genotype (%):**			**				n.s.	
1	57.1	57.1		57.1	59.2	59.0		59.2
2	8.0	6.6		7.3	8.1	6.9		7.5
3	31.7	32.6		32.1	29.3	30.5		29.8
4	2.7	3.4		3.0	2.8	3.3		3.1
5	0.2	0.1		0.2	0.3	0.1		0.2
6	0.3	0.2		0.2	0.3	0.2		0.2
**High baseline viral load (> 470.000 IU/ml, %)**	52.9	55.8	**	54.4	52.8	56.7	***	54.7
**RVR (%)**	43.7	43.0	n.s.	43.3	43.6	41.2	n.s.	42.1
**SVR total (%)**	50.3	50.5	n.s.	50.4	62.0	63.7	n.s.	62.8
SVR genotype 1 (%)	43.7	45.5	n.s.	44.6	52.0	55.6	**	53.7
SVR genotype 2 (%)	60.8	62.9	n.s.	61.7	73.9	76.2	n.s.	74.9
SVR genotype 3 (%)	59.4	57.4	n.s.	58.4	79.3	77.6	n.s.	78.5
SVR genotype 4 (%)	48.3	41.8	n.s.	44.8	56.9	54.4	n.s.	55.6
SVR genotype 5 (%)	62.5	62.5	n.s.	62.5	66.7	71.4	n.s.	68.2
SVR genotype 6 (%)	64.7	70.0	n.s.	66.7	78.6	77.8	n.s.	78.3
**Treatment discontinuation (%)** ^‡^	23.1	27.2	***	25.1	17.4	17.9	n.s.	17.6
Virological nonresponse	9.1	10.4	*	9.7	11.2	13.1	**	12.1
Poor tolerability	5.1	3.1	***	4.2	6.3	4.0	***	5.2
Other reasons	10.2	14.9	***	12.5	1.5	2.5	***	2.0
Death	0.1	0.3	**	0.2	0.1	0.3	**	0.2
**Mean treatment duration (weeks)**	33.3	32.8	n.s.	33.1	35.0	35.0	n.s.	35.0
**Mean ribavirin dosage (mg/kg)**	11.1	11.8	***	11.5	11.6	12.5	***	12.0
Genotype 1	10.9	11.2	**	11.1	11.3	11.9	***	11.6
Genotype 2	11.3	13.0	***	12.0	11.8	13.5	***	12.6
Genotype 3	11.5	12.7	***	12.1	12.2	13.5	***	12.8
Genotype 4	10.5	10.6	n.s.	10.5	10.8	11.2	n.s.	11.0
Genotype 5	11.3	11.4	n.s.	11.3	11.6	13.0	n.s.	12.0
Genotype 6	12.3	13.2	n.s.	12.6	12.9	13.8	n.s.	13.2

^†^Data on ethnicity were not collected in 2003. Hence, no data on ethnicity are available for the very early era of HCV treatment. There is no indication that the composition of ethnicity in 2003 is different from that in the following years (2004–2007)

^‡^ multiple answers possible

APRI, aspartate aminotransferase (AST) platelet ratio index; BMI, body mass index; GGT, gamma-glutamyl transferase; n.s., not significant; RVR, rapid virological response; SVR, sustained virological response

*p < 0.05;

** p < 0.01,

*** p < 0.001

A significantly higher proportion of patients in the later treatment period had comorbidities than in the earlier period (Table 1). Therefore, a matched pair analysis was performed to control for bias related to this observation.

### Matched-pair analysis

A total of 2,997 matched pairs remained for the MP-PP cohort. The majority of patients was infected with genotype 1 (n = 1,946), followed by patients infected with genotype 3 (n = 861). Lesser numbers of patients were infected with genotypes 2 (n = 147) and genotype 4 (n = 43). Demographic data are shown in Table 2.

**Table 2:** Baseline demographics, virological responses, treatment discontinuations as well as treatment duration and mean ribavirin dose in two periods of 2003–2007 and 2008–2011 in the matched pairs PP and matched pairs PP completer population.

	Matched pairs PP				Matched pairs PP completers			
	2003–2007	2008–2011	p-value	total	2003–2007	2008–2011	p-value	total
**Number of patients (n)**	2997	2997		5994	2439	2475		5994
**Age (Yrs, mean)**	42.4	42.6	n.s.	42.5	41.3	41.6	n.s.	42.5
**Gender (male, %)**	61.0	61.0	n.s.	61.0	61.5	61.1	n.s.	61.0
**BMI (kg/m2, mean)**	25.2	25.6	***	25.4	25.1	25.5	**	25.4
**Ethnicity, Caucasian (%; adjusted by unknown)** ^†^	76.0	96.9	n.s.	86.5	76.7	96.9	n.s.	86.5
**APRI Score (%)**			n.s.				n.s.	
< 0.5	46.6	46.6		46.6	48.8	48.7		46.6
0.5-< = 1.5	43.8	43.8		43.8	44.2	44.1		43.8
1.5-< = 2	2.4	2.4		2.4	2.0	1.9		2.4
>2	7.2	7.2		7.2	4.9	5.3		7.2
**Baseline GGT (IU/ml, mean)**	80	90	n.s.	85	70	74	n.s.	85
**Route of infection (%):**			n.s.				n.s.	
Transfusion/blood products	12.1	12.1		12.1	11.1	11.1		12.1
Intravenous drug abuse	38.5	38.5		38.5	41.0	41.3		38.5
Other (unknown)	49.4	49.4		49.4	47.9	47.6		49.4
**Concomitant diseases (%):**	49.6	49.6	n.s.	49.6	48.6	47.8	n.s.	49.6
Psychiatric	9.5	12.7	***	11.1	9.2	12.4	***	11.1
Cardiovascular	8.2	8.8	n.s.	8.5	7.3	7.5	n.s.	8.5
Diabetes	4.1	3.9	n.s.	4.0	3.2	3.1	n.s.	4.0
Pulmonary	3.3	2.7	n.s.	3.0	3.0	2.8	n.s.	3.0
Former iv drug abuse	17.5	20.0	*	18.7	18.9	21.4	*	18.7
Opioid substitution treatment	8.6	8.6	n.s.	8.6	9.4	9.2	n.s.	8.6
**Genotype (%):**			n.s.				n.s.	
1	64.9	64.9		64.9	59.7	59.8		64.9
2	4.9	4.9		4.9	5.7	5.7		4.9
3	28.7	28.7		28.7	33.3	33.2		28.7
4	1.4	1.4		1.4	1.3	1.3		1.4
**High baseline viral load (> 470,000 IU/ml, %)**	55.8	55.8	n.s.	55.8	53.9	53.3	n.s.	55.8
**RVR (%)**	40.8	40.2	n.s.	40.4	48.4	46.4	n.s.	40.4
**SVR total (%)**	61.2	64.3	*	62.7	74.3	77.9	**	62.7
SVR genotype 1 (%)	51.9	56.3	**	54.1	68.5	73.9	**	54.1
SVR genotype 2 (%)	71.4	75.5	n.s.	73.5	74.8	79.3	n.s.	73.5
SVR genotype 3 (%)	80.5	81.0	n.s.	80.7	84.4	84.8	n.s.	80.7
SVR genotype 4 (%)	58.1	55.8	n.s.	57.0	77.4	75.0	n.s.	57.0
**Treatment discontinuation (%)** ^‡^	17.8	17.4	n.s.	17.6	0.0	0.0		17.6
Virological nonresponse	11.8	13.0	n.s.	12.4				12.4
Poor tolerability	6.3	3.6	***	4.9				4.9
Other reasons	1.5	2.4	*	2.0				2.0
Death	0.0	0.3	**	0.2				0.2
**Mean treatment duration (weeks)**	35.8	35.8	n.s.	35.8	39.0	38.6	n.s.	35.8
**Mean ribavirin dosage (mg/kg)**	11.6	12.6	***	12.1	12.8	13.8	***	12.1
Genotype 1	11.4	12.1	***	11.7	13.2	13.8	***	11.7
Genotype 2	11.5	13.3	**	12.4	11.8	13.4	**	12.4
Genotype 3	12.0	13.5	***	12.8	12.2	13.7	***	12.8
Genotype 4	10.1	11.8	n.s	11.0	12.2	13.6	n.s.	11.0

^†^Data on ethnicity were not collected in 2003. Hence, no data on ethnicity are available for the very early era of HCV treatment. There is no indication that the composition of ethnicity in 2003 is different from that in the following years (2004–2007)

^‡^ multiple answers possible

APRI, aspartate aminotransferase (AST) platelet ratio index; BMI, body mass index; GGT, gamma-glutamyl transferase; n.s., not significant; RVR, rapid virological response; SVR, sustained virological response

*p < 0.05;

** p < 0.01,

*** p < 0.001

The overall SVR rates were significantly higher in the later than in the earlier period (64.3% versus 61.2%; p = 0.008). The SVR rates in patients with genotype 1 infection were 51.9% in the earlier years versus 56.3% in the later years (p = 0.0057). For genotype 3 infected patients SVR rates showed no statistical difference (80.5% versus 81.0%, p = 0.8070). For genotype 2 infected patients, SVR rates were 71.4% in the earlier years versus 75.5% in the later years (p = 0.5091).

Improved SVR rates were observed throughout all relevant subgroups, namely those with favorable and those with unfavorable baseline characteristics. Patients below the age of 45 had response rates of 71.8% in 2003–2007 and 75.2% in 2008–2011, respectively (p = 0.0296), and patients aged 45 or older had response rates of 47.1% in 2003–2007 and 50.8% in 2008–2011, respectively (p = 0.0618). SVR rates in patients with low viral load was 70.2% and 72.3%, respectively, while patients with high viral load showed response rates of 54.0% vs. 58.0%, respectively (p = 0.0179). In patients with normal baseline gamma-GT, response rates were 72.0% in the earlier years and 74.6 in the later years (p = 0.0884), whereas patients with elevated gamma-GT showed response rates of 48.3% in 2003–2007 and of 52.1% in 2008–2011 (p = 0.0469).

By univariate testing, factors other than the treatment period were significant predictors of treatment success. The HCV genotype (p<0.0001) as well as viral load (p<0.0001) were significantly associated with SVR as were patient age (p<0.0001) and possible route of infection (p<0.0001). Presence of concomitant disease (p = 0.002), substitution for opioid abuse (p<0.0001), and elevated gamma-GT (p = 0.0001) as well as higher APRI score (p<0.0001) had a negative impact on treatment outcome. The duration of treatment was inversely associated with success rates (p<0.0001). This apparent paradox is explained by the selection of patients with unfavorable baseline characteristics such as higher age, BMI, GGT, proportion of patients with high baseline viral load and on-treatment parameters for longer treatment duration (data not shown). For example, the proportion of patients with APRI score > 1.5 was 5.7% in patients with shorter treatment duration compared with 10.3% in patients with longer treatment duration, which may also contribute to this explanation. The dose of ribavirin showed a weak, borderline statistically significant, association with treatment response (p = 0.046).

#### Effect of individualization of treatment duration on treatment efficacy

**Fig 2:**
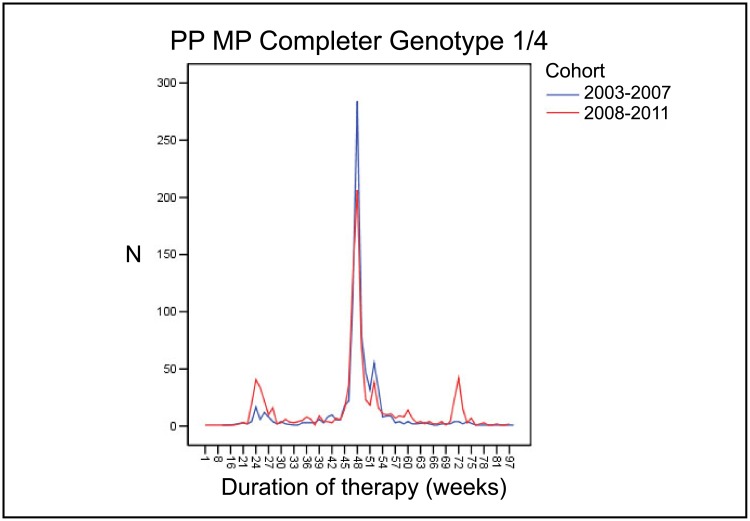
Comparison of treatment duration in patients infected with genotype 1/4 between 2003–2007 and 2008–2011 period. MP, matched pairs; PP, per protocol.

**Fig 3:**
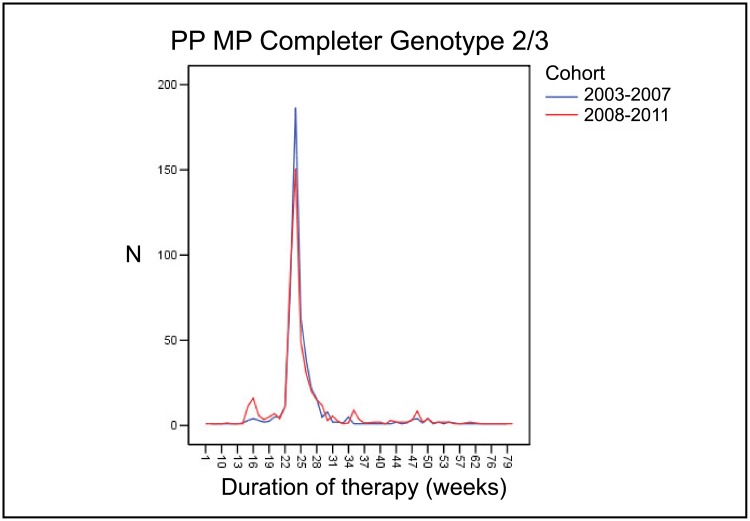
Comparison of treatment duration in patients infected with genotype 2/3 between 2003–2007 and 2008–2011 period. MP, matched pairs; PP, per protocol.

To evaluate the effect of individualization of treatment regimens between the earlier and the later period, we analyzed the MP-PP completer cohort regarding planned treatment durations according to genotype. The mean treatment duration was 35.8 weeks for all patients in both periods. However, treatment duration was significantly more heterogeneous in the later period. In genotype 1/4 patients, the standard treatment duration of 48 weeks was chosen in 81.2% of patients who completed the treatment in the years 2003–2007, while only 64.6% of patients who completed the treatment were treated for 48 weeks in 2008–2011. Treatment was abbreviated in more patients in the later than in the earlier period (19.2% versus 9.3% of patients who completed the treatment; Fig 2). Likewise, treatment extension was more frequent in the later than in the earlier period (16.1% versus 9.5% of patients who completed the treatment). The same effect was observed in genotype 2/3 patients, in whom the standard treatment duration is 24 weeks. 87.2% of patients who completed the treatment were treated for 24 weeks in the years 2003–2007 while only 75.6% of patients who completed the treatment were treated for 24 weeks in the years 2008–2011. In contrast, more patients were treated for a shorter duration in the later period (10.0% of patients who completed the treatment) than in the earlier period (3.0% of patients who completed the treatment; Fig 3). Again, treatment was also extended in more patients in the later period. In 2008–2011, treatment durations beyond 28 weeks were observed in 14.4% of patients who completed the treatment while only 9.8% of patients who completed the treatment were treated longer in the years 2003–2007.

Of the patients in the MP-PP completer cohort with favorable predictive factors (APRI < 0.5, low baseline viral load (<800.000 IU/ml), RVR) who qualified for the shortening of treatment in principle, the percentage of patients who actually received abbreviated treatment courses increased between the earlier and the later period. 27.7% of genotype 1/4 infected patients received treatment for less than 44 weeks in the earlier period compared to 59.3% in the later period. Likewise, the percentage of eligible patients with genotype 2/3 infection receiving less than 20 weeks of therapy rose from 4.8% to 25.3%.

#### Effect of individualization of ribavirin dosing on treatment efficacy

We analyzed whether prescription of ribavirin followed a more individualized regimen in the later period than in the earlier period, thus reflecting dosing according to actual weight rather than the categories put forward in the prescription information. The mean ribavirin dose was 11.6 mg/kg in the 2003–2007 period and 12.6 mg/kg in the 2008–2011 period in the MP-PP cohort (p<0.001, OR = 1.051, CI 95%:1.035–1.067). In patients achieving an SVR, mean ribavirin doses were 12.7 mg/kg and 13.7 mg/kg in the earlier and the later period, respectively. The percentage of patients receiving a ribavirin dose exceeding 13.2 mg/kg was 44.5% in the earlier period and 56.0% in the later period. The latter analysis was restricted to patients who reached a planned treatment stop since cumulative ribavirin doses are not meaningful when treatment was stopped prematurely.

#### Multivariate analysis

Multivariate analysis was performed in the MP-PP completer cohort. SVR rates between the two treatment periods remained significantly different (p = 0.002, OR = 1.254, CI 95%:1.089–1.444). Virus genotype 3 vs. 1 (p<0.0001, OR = 1.851, CI 95%: 1.549–2.212) and low baseline viral load (p<0.0001, OR = 1.567, CI 95%: 1.355–1.813) predicted treatment outcome. Younger patient age (p<0.0001, OR = 2.063, CI 95%: 1.773–2.399), normal gamma-GT values (p<0.0001, OR = 1.528, CI 95%: 1.312–1.779), and female gender (p<0.010, OR = 1.238, CI 95%: 1.065–1.440) as well as lower APRI score (0.028, OR = 1.533, CI 95%: 1.128–2.084) and higher ribavirin dose (p = 0.032, OR = 1.024, CI: 0.861–1.219) were significantly associated with success rates. Treatment duration remained inversely correlated with treatment outcomes, with lower SVR rates in patients treated for longer durations.

## Discussion

Treatment of chronic HCV infection with pegIFN alfa and ribavirin has been the standard of care for many years [5]. Individualization of this treatment increased outcomes in many randomized controlled trials and allowed patients to shorten treatment without compromising efficacy, thus saving costs and sparing the patients side effects [12]. However, it is unknown whether treatment individualization was implemented by the majority of physicians who treated patients in office-based practices, and whether individualization of treatment conferred any benefit regarding treatment outcomes in the real life setting.

The large noninterventional cohort study presented here shows that individualization of HCV treatment was indeed more common in the 2008–2011 period than in the 2003–2007 period, i.e. after individualization was included as a recommendation into national and international treatment guidelines [30, 32]. In the present study treatment durations other than 24 weeks for genotypes 2/3 and other than 48 weeks for genotypes 1/4 as well as the dosing of ribavirin were analyzed as indicators for individualization between the two treatment periods. However, SVR rates in both treatment periods did not differ significantly (overall SVR rates were 62.0% and 63.7%, respectively). The effect on treatment outcomes was most likely confounded by the fact that treatment had been extended to sicker patients in the later period. Indeed, the proportion of patients with concomitant diseases was 44.3% in the earlier period as compared to 57.1% in the later period.

To allow analysis despite this confounding factor, we performed a matched-pair analysis, in which patients were matched for baseline factors including To allow analysis despite this confounding factor, we performed a matched-pair analysis, in which patients were matched for the following baseline factors known to influence treatment outcome: age (range of 5 years), HCV genotype, baseline viral load (low or high viral load with best cut-off of ≤ 470.000 IE/ml), APRI-Score (< 0.5, 0.5–< = 1.5, 1.5–< = 2, > 2), possible route of infection (intravenous drug abuse, blood or blood products, unknown), presence or absence of concomitant diseases, baseline gamma-GT (within normal range versus elevated), and substitution for intravenous drug abuse. (10). Strikingly, after matching for these confounders, SVR rates in the 2008–2011 period were significantly higher than in the earlier period. SVR rates improved in both easier to treat as well as more difficult to treat patient populations. This finding suggests that physicians became better acquainted with treatment regimens and the management of side effects, allowing more patients to remain on treatment or uphold the assigned doses of pegIFN alfa and ribavirin.

As an indicator for individualization, the number of patients who received their treatment for durations other than 24 weeks for genotypes 2/3 and 48 weeks for genotypes 1/4 (reviewed in [12]) was higher in the 2008–2011 period than in the 2003–2007 period. More patients were treated with an abbreviated regimen of 24 weeks in genotype 1/4 infection and 16 weeks in genotype 2/3 patients without compromising SVR rates. While 81.2% of patients infected with HCV genotypes 1/4 in the earlier period received treatment for the standard duration of 48 weeks, this number dropped to 64.6% in the later period. The same difference was observed in patients with genotype 2/3 infection, in which 87.2% and 75.6% of patients received the standard treatment of 24 weeks in the earlier and later period, respectively. The difference was due to both, higher numbers of patients receiving abbreviated regimens in the later than in the earlier years (19.2% versus 9.3% in patients with genotype 1/4 infection and 10.0% versus 3.0% in patients with genotype 2/3 infection) and higher numbers of patients receiving extended treatments (16.1% versus 9.5% in patients with genotype 1/4 infection and 14.4% versus 9.8% in patients with genotype 2/3 infection).

When only those genotype 1/4 patients who were eligible for treatment abbreviation in principle were considered, the proportion of patients who indeed received less than 48 weeks of therapy doubled in the later period from 27.7% to 59.3%. While this increase is substantial, individualization of treatment duration was forgone in approximately 40% of eligible patients. A previous prospective clinical trial demonstrated that 29% of treatment naive HCV patients infected with genotype 1/4 were eligible for abbreviation of treatment duration, likewise indicating that individualization of treatment durations was not yet commonplace in daily practice in Germany [18].

The second indicator for treatment individualization which could contribute to better SVR rates in the later period is the adjustment of ribavirin dosing to the actual body weight rather than to the categories of body weight suggested in the label, as proposed in earlier clinical trials [33, 34]. Despite the label, which sets the ribavirin dose at 1200 mg for patients weighing 75 kg or more and at 1000 mg in patients weighing less than 75 kg in genotype 1 and 4 infected patients and at 800 mg flat for all patients infected with genotypes 2 or 3, recent data suggests that this dose is insufficient for some patients, especially those which receive less than 13 mg/kg of ribavirin in genotypes 1/4 and less than 10 mg/kg in genotypes 2/3 [33]. We observed that higher ribavirin doses were prescribed in the later than in the earlier period. The mean ribavirin dose per kg was 1 mg higher in the later than in the earlier period in all patients as well as in patients achieving an SVR. However, the ribavirin dose was higher to a similar extent in patients not achieving an SVR, suggesting that increased ribavirin dosing was at least not the only factor responsible for the improvement of SVR rates. The multivariate analysis of factors associated with SVR in the MP-PP completer cohort showed that treatment in the later period, younger age, female gender, HCV genotype non-1/4, lower baseline viral load, lower APRI score, normal gamma-GT, longer treatment duration, and higher ribavirin dosing were independently associated with treatment success. These data underline the importance of treatment individualization, which adds to well-established predictors in the real world setting.

Are the results from the present study still relevant in the beginning era of interferon-free treatment regimens [35]? While a relatively liberal access to DAAs is anticipated in western countries, many parts of the world will not easily implement interferon-free treatment regimens given their excessive costs [36]. Therefore, pegIFN alfa will possibly remain a part of the standard of care until access strategies for DAAs in lower-income countries have been initiated. Treatment individualization is cost-effective [37], and the factors identified here in a large cohort of patients treated with pegIFN alfa-2a and ribavirin may still apply to patients treated with triple regimens, although individualization of treatment durations is largely offset by the mandatory response-guided treatment approaches in boceprevir- and telaprevir-based triple treatment [38] and by the fixed treatment durations mandated in simeprevir- and sofosbuvir-based triple treatment [39, 40].

This study was an observational study, which gave the participating physicians liberty to decide on treatment duration as well as on dosing. Naturally, data from an observational study are more susceptible to bias resulting from poorer quality of data. On the contrary, only a real-life study allows the observation of treatment effects in a population that is not heavily selected for homogeneity and excludes problematic patients, as is typically the case in randomized controlled studies. We therefore believe that the current study is a valuable addition to the data generated in randomized controlled studies by providing data from a sample size unaffordable in controlled studies and including patients with unfavorable baseline factors, which may be deemed unsuitable for participation in controlled studies. The limitation of our study is the absence of a mandatory treatment protocol, leaving space for deviation from the treatment regimens recommended in guidelines. However, we believe that the very large sample size, which to our knowledge is the largest in any observational study in hepatitis C treatment, levels the potential bias and is the biggest asset of this study.

In summary, our data show that individualization of HCV therapy was cautiously implemented in daily practice among patients treated for chronic hepatitis C infection in Germany. Indicators for individualization included heterogenous treatment durations as well as higher ribavirin doses. Treatment individualization resulted in higher SVR rates comparable to those observed in randomized controlled trials.

## Supporting Information

S1 Study Protocol Study Protocol. (DOC)
